# Four Loci Are Associated with Cardiorespiratory Fitness and Endurance Performance in Young Chinese Females

**DOI:** 10.1038/s41598-020-67045-y

**Published:** 2020-06-22

**Authors:** Ying Zhao, Guoyuan Huang, Zuosong Chen, Xiang Fan, Tao Huang, Jinsheng Liu, Qing Zhang, Jingyi Shen, Zhiqiang Li, Yongyong Shi

**Affiliations:** 1 Physical Education Department, Shanghai Jiao Tong University, Shanghai, 200240 China; 2 Pott College of Science, Engineering and Education, University of Southern Indiana, Indiana, 47712 USA; 3 School Infirmary, Shanghai Jiao Tong University, Shanghai, 200240 China; 4 Bio-X Institutes, Key Laboratory for the Genetics of Developmental and Neuropsychiatric Disorders (Ministry of Education), Collaborative Innovation Center for Brain Science, Shanghai Jiao Tong University, Shanghai, 200240 China; 5 Affiliated Hospital of Qingdao University, Qingdao, 266003 China; 6 Biomedical Sciences Institute of Qingdao University (Qingdao Branch of SJTU Bio-X Institutes), Qingdao University, Qingdao, 266003 China; 7 Shanghai Key Laboratory of Psychotic Disorders, Shanghai Mental Health Center, Shanghai Jiao Tong University School of Medicine, Shanghai, 200030 China; 8 Department of Psychiatry, First Teaching Hospital of Xinjiang Medical University, Urumqi, 830046 China

**Keywords:** Genetics, Biomarkers, Risk factors

## Abstract

Cardiorespiratory fitness (CRF) and endurance performance are characterized by a complex genetic trait with high heritability. Although research has identified many physiological and environmental correlates with CRF, the genetic architecture contributing to CRF remains unclear, especially in non-athlete population. A total of 762 Chinese young female participants were recruited and an endurance run test was used to determine CRF. We used a fixed model of genome-wide association studies (GWAS) for CRF. Genotyping was performed using the Affymetrix Axiom and illumina 1 M arrays. After quality control and imputation, a linear regression-based association analysis was conducted using a total of 5,149,327 variants. Four loci associated with CRF were identified to reach genome-wide significance (P < 5.0 × 10^-8^), which located in 15q21.3 (rs17240160, P = 1.73 × 10^-9^, GCOM1), 3q25.31 (rs819865, P = 8.56 × 10^-9^, GMPS), 21q22.3 (rs117828698, P = 9.59 × 10^-9^, COL18A1), and 17q24.2 (rs79806428, P = 3.85 × 10^-8^, PRKCA). These loci (GCOM1, GMPS, COL18A1 and PRKCA) associated with cardiorespiratory fitness and endurance performance in Chinese non-athlete young females. Our results suggest that these gene polymorphisms provide further genetic evidence for the polygenetic nature of cardiorespiratory endurance and be used as genetic biomarkers for future research.

## Introduction

Cardiorespiratory fitness (CRF) is considered a core component of health-related fitness. Over the past 30 years, a growing body of research has examined factors associated with CRF. Many biological, social, and environmental factors have been identified to be correlated to CRF^1,2^. Indexed by maximal oxygen consumption (VO_2max_), CRF is closely associated with sports and athletic performance. CRF is an important determinant particularly for endurance athletes. Endurance performance is a complex phenotype subject to the influence of both environmental and genetic factors. For example, some somatotypes, such as height and body type, are classically considered to be associated with power or endurance athlete status, respectively^3^.

It has been accepted in the past decade that genetic traits are strongly associated with human physical performance. Genetic polymorphism is suggested to be associated with CRF and endurance performance. Studies on families and twins indicated that CRF was genetically determined in part with heritability estimates about 50% and ranging between 25% and 65%^4,5^. It was suggested that the heritability of athlete status in British female was estimated at approximately 66%^6^. A group of single nucleotide polymorphisms (SNPs) were identified to be related to aerobic capacity, endurance and increases in VO_2max_. Amir *et al*.^7^ examined an angiotensin-converting enzyme (ACE) insertion/deletion (I/D) polymorphism. They found a higher number of D-allele carriers and D/D genotypes in endurance athletes than in sedentary controls. In the peroxisome proliferator-activated receptor alpha (PPARɑ) gene, significant different genotype frequencies were reported for the intron 7 G/C genetic variant between endurance and power oriented athletes and non-athlete control^8^. In a study on the Arg16Gly polymorphism, an excess of Gly-allele carriers was found in sedentary controls, suggesting that Gly-allele is negatively correlated with performance status of endurance athlete^9^. Other data showed that the NFIA-AS2 rs1572312, TSHR rs7144481 and RBFOX1 rs7191721 polymorphisms were associated with aerobic performance and elite endurance athlete status^10^. Yoo *et al*.^11^ reported that 7 SNPs were identified to predict gains in VO_2max_ that accounted for 26.0% of the variance in the increment of VO_2max_. The ACE insertion/deletion (ACE I/D, rs1799752) polymorphism has been related with improvements in performance and exercise duration in a variety of populations. In a Systematic Review and Meta-Analysis, more solid evidence was found for the associations between ACE II genotype and endurance events^12^. However, a multi-cohort quantitative analysis, as well as some other case-control studies, demonstrated that ACTN3 XX genotype and ACE II genotype were unlikely to provide an advantage in competitive endurance running performance^13^.

Taken together, the last decade has seen a variety of specific genetic factors proposed. However, each is likely to make a limited contribution to intermediary traits of fitness and an ‘elite’ phenotype. It seems more likely that such status depends on the simultaneous presence of multiple such variants^14^. Noticeably, it is clear that the current use of genetic tests for the prediction of future elite athlete status is ineffectual, a finding that echoes recent consensus statements^15,16^. Despite the increasing availability of commercial genetic tests, the currently available data suggest the use of the information provided by these tests for talent identification or selection is unfounded; in fact, a far greater number of performance-enhancing polymorphisms need to be both discovered and replicated in subsequent studies^17^, including research on different non-athletic populations. Further, identification of novel genetic biomarkers associated with CRF and endurance potential is clinically meaningful and can help reduce the risk of cardiovascular disease and all-cause death. To date, the genome-wide association study (GWAS) on cardiorespiratory fitness and endurance performance for Chinese non-athlete young females is still lacking. Based on these considerations, we hypothesized there were CRF-related genetic variants in some genetic loci that possibly indicated the polygenetic nature of CRF and aerobic performance. By using GWAS approach, this study aimed to investigate the association between multiple single nucleotide polymorphisms (SNPs) and traits of cardiorespiratory fitness and endurance performance in Chinese non-athlete female individuals.

## Results

### Study subject characteristics and running performance

A total of 762 subjects were included in the analyses and final results. The main characteristics of the studied subjects are shown in Table 1.

**Table 1:** The main characteristics of subjects.

N	Height (cm)	Weight (kg)	BMI (kg.m^-2^)	800-m running (s)
762	161.77 ± 5.25	54.12 ± 7.08	20.67 ± 2.41	238.3 ± 28.76

*Note*: Values are means ± SD. Abbreviations as BMI = body mass index.

### Genome-wide analyses

**Figure 1:**
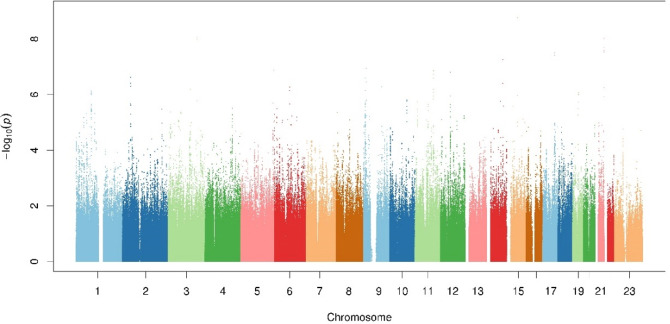
The Manhattan plot of the 800-meter running performance.

The results for the associations between CRF and the loci identified in the GWAS were presented in Table 2, showing these significant and suggestive SNPs. In female college students, significant associations were detected between genotype and 800-meter running test time. The Manhattan plot of the 800-meter running performance is displayed in Fig. 1. The QQ plot for the P value distribution showed that the observed P values departed from the expected P values only at the right end tail of the distribution (Supplemental Fig. S1). The genomic inflation factor (λ) of the meta-analysis was 1.005.

**Table 2:** Results for the association of CRF and loci identified in the GWAS.

Info	Effect/Another Allele	Phase	MAF	BETA (95% CI)	P value
***Significant SNPs***					
rs17240160	A/T	Data set 1	0.042	26.9(14.7–39.1)	2.10E-05
Chr15:58078261		Data set 2	0.023	25.4 (13.5–37.2)	3.28E-05
*GCOM1*		Combined		26.1	1.73E-09
rs819865	G/A	Data set 1	0.064	13.8 (4.2–23.4)	4.94E-03
Chr3:155716421		Data set 2	0.057	18.7 (11.5–25.9)	5.69E-07
*GMPS*		Combined		16.9	8.56E-09
rs117828698	A/C	Data set 1	0.044	14.5 (2.9–26.1)	1.44E-02
Chr21:46849501		Data set 2	0.038	24.1 (15.3–32.9)	1.48E-07
*COL18A1*		Combined		20.6	9.59E-09
rs79806428	C/T	Data set 1	0.072	23.3 (14.5–32.1)	3.71E-07
Chr17: 66282767		Data set 2	0.049	11.3 (2.9–19.7)	8.59E-03
*PRKCA*		Combined		17.0	3.85E-08
***Suggestive SNPs***					
rs12518860	A/G	Data set 1	0.039	15.9 (3.4–28.5)	1.35E-02
Chr5:178208844		Data set 2	0.033	23.9 (14.1–33.8)	2.68E-06
*AACSP1*		Combined		20.9	1.32E-07
rs1384206	G/T	Data set 1	0.065	12.0 (2.1–21.9)	1.85E-02
Chr15:58078261		Data set 2	0.061	17.5 (10.4–24.7)	2.26E-06
*CNTN5*		Combined		15.6	1.36E-07
rs941138	C/T	Data set 1	0.158	14.2 (7.7–20.6)	2.09E-05
Chr:12:53614349		Data set 2	0.185	8.0 (3.3–12.6)	8.97E-04
*RARG*		Combined		10.1	1.58E-07
rs1951850	C/T	Data set 1	0.202	8.1 (2.3–13.9)	6.66E-03
Chr9:9858194		Data set 2	0.190	9.8 (5.4–14.2)	1.39E-05
*PTPRD*		Combined		9.2	2.54E-07

*Note*: Abbreviations as Info = SNP informations; BETA = Regression coefficient; 95% CI = 95% confidence intervals; Chr = Chromosome; MAF = Minor allele frequency.

**Figure 2:**
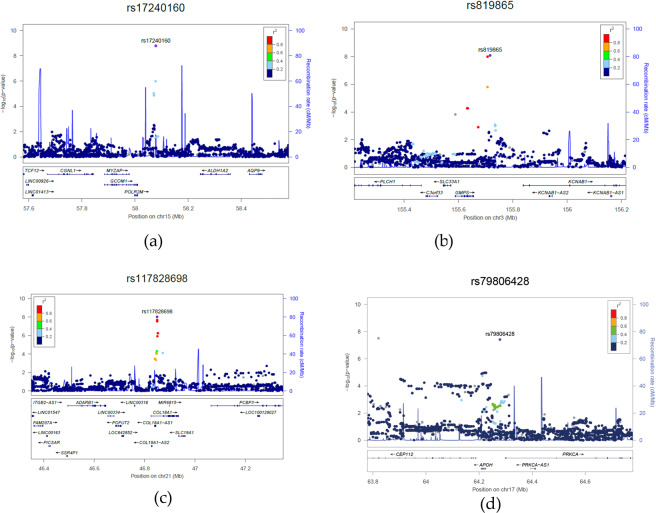
Regional association plots of loci associated with CRF. (**a**) rs17240160, (**b**) rs819865, (**c**) rs117828698 and (**d**) rs79806428. Purple circles represent the most significantly associated SNP (marker SNP) in each region based on the meta-analysis of discovery and replication. ‒log10 P values (y axis) of SNPs (within regions spanning 500 kb on either side of the marker SNP) are presented according to their chromosomal positions (x axis, hg19). SNPs are colored according to their linkage disequilibrium (LD) with the marker SNP. The LD values were established based on the 1000 Genome Asian (ASI) data (March 2012) and shown in table S2. Estimated recombination rates with samples from the 1000 Genomes Project (March 2012 release) are shown as blue lines, and the genomic locations of genes within the regions of interest annotated from the UCSC Genome Browser, are shown as arrows.

The meta-analysis revealed four loci that reached the genome-wide significance level of *P* < 5 × 10^-8^, including the loci within *GCOM1* (Fig. 2a), *GMPS* (Fig. 2b), *COL18A1* (Fig. 2c), and *PRKCA* (Fig. 2d). There was no evidence for heterogeneity between the data sets for these variants (P value for Cochran’s Q test> 0.05) (Supplemental Table S1). The most significant SNPs to be associated with CRF and endurance performance were located in *GCOM1* (rs17240160, 15q21.3) with a P value of 1.73 × 10^−9^. Figure 2a shows the significant association of CRF for the A/T allele of the GCOM1 rs17240160 polymorphism (*P* = 1.73 × 10^−9^). There was a significant association with CRF for the subjects with the G/A genotype of the GMPS rs819865 (*P* = 8.56 ×10^−9^) (Fig. 2b). The A/C allele of the COL18A1 rs117828698 polymorphism was significantly associated with CRF (*P* = 9.59 ×10^−9^) (Fig. 2c). Subjects with the C/T genotype of the PRKCA rs79806428 polymorphism showed significant association with CRF and endurance performance (*P* = 3.85 ×10^−8^) (Fig. 2d). Table 2 additionally presented four suggestive loci that did not reach the genome-wide significance threshold but showed *P* values <5 × 10^-7^. Four SNPs were the genotypes of the AACSP1 rs12518860 A/G (*P* = 1.32 ×10^−7^), the CNTN5 rs1384206 G/T (*P* = 1.36 ×10^−7^), the RARG rs941138 C/T (*P* = 1.58×10^−7^), and the PTPRD rs1951850 C/T (*P* = 2.54 ×10^−7^).

### Functional implications of the identified loci

HaploReg^28^ was used to explore the functional implications for the associated loci. It included the chromatin state and protein binding annotation from the Roadmap Epigenomics and ENCODE projects, effects on regulatory motifs, expression from eQTL studies, and others. Table 3 shows functional annotations for CRF-endurance performance-associated SNPs. Specifically, the GCOM1 rs17240160 polymorphism was located in the H3K4me1 marked enhancer regions. It involves in 8 tissues including the heart (right atrium and aorta), lung, and brain (hippocampus middle, substantia nigra, cingulate gyrus, and angular gyrus). The COL18A1 rs117828698 polymorphism was co-located in potential regulation regions that were indicated as promoter and enhancer marks. Many related tissues of the COL18A1 rs117828698 polymorphism include the heart, lung, brain, and muscle (Supplementary Table S3). The RARG rs941138 polymorphism was located in a conserved region as indicated by GERP and SiPhy. The region is predicted in the active chromatin state in all the tested cells/tissues. In addition, the GMPS rs819865, COL18A1 rs117828698, and PRKCA rs79806428 polymorphism were found to be the cis-regulation SNPs in the Genotype-Tissue Expression pilot analysis (Table 3).

**Table 3:** Functional annotations for CRF-associated SNPs.

Variant	Promoter histone marks	Enhancer histone marks	DNAse	Proteins bound	Motifs changed	Selected eQTL hits	RefSeq genes	dbSNP func annot
rs17240160	ESC	8 tissues			4 altered motifs		GCOM1	
rs819865					CEBPD	1 hit	GMPS	
rs117828698	8 tissues	17 tissues	4 tissues		4 altered motifs	1 hit	COL18A1	intronic
rs79806428		8 tissues			Zbtb3	1 hit	PRKCA	
rs12518860	ESDR, SPLN	10 tissues			4 altered motifs		AACSP1	
rs1384206					AP-1		CNTN5	
rs941138	24 tissues	21 tissues	47 tissues	4 bound proteins	CEBPB,Pou2f2		RARG	intronic
rs1951850	PANC	PANC, PLCNT			6 altered motifs		PTPRD	intronic

*Note:* Evidence of histone marks, DNAse hypersensitivity sites or transcription factor occupancy using HaploReg v4 are shown for the CRF-associated SNPs (both genome-wide significance and suggestive evidence).

For details on data sources and abbreviations, see the full documentation of HaploReg v4 (https://pubs.broadinstitute.org/mammals/haploreg/haploreg.php).

## Discussion

This was the first GWAS to assess genetic determinants of CRF in non-athlete female young individuals of Chinese Han ethnicity. The results of this study revealed: 1) four GWAS loci (GCOM1, GMPS, COL18A1, and PRKCA) were associate with CRF and endurance performance that reached the genome-wide significance threshold (*P* < 5 × 10^-8^) and 2) four additional loci (AACSP1, CNTN5, RARG, PTPRD) were identified with suggestive evidence (*P* < 5×10^-7^).

Of the discovered SNPs, the GCOM1 rs17240160 polymorphism showed the most significantly higher association with 800-metre running performance in our study. The human GCOM1 complex gene is involved in transcription elongation. It is associated with the intercalated discs of cardiomyocytes in relation to multiple heart diseases. A recent study demonstrated that GCOM1 also plays a role in regulating neuroprotection^29^. In the present report, we found that the GCOM1 rs17240160 polymorphism was significantly associated with cardiorespiratory endurance performance. Specifically, the young female college students with the A/T genotype showed significantly higher association with the 800-meter running performance. Considering the previous and present findings, it is possible that the GCOM1 rs17240160 polymorphism results in increased activities of cardiovascular and neural protections, which is a possible physiological mechanism underlying the relation between the GCOM1 rs17240160 polymorphism and CRF and endurance performance.

The rs819865 SNP is located near the GMPS gene. This gene is a member of the G-type aminotransferase family. The GMPS catalyzes XMP amination to produce GMP in the de novo synthesis of guanine nucleotides. In the catalytic process, glutamine is hydrolyzed to form the amino group required for the amination reaction. In the presence of magnesium ions, ATP drives the reaction^30^. The GMPS specifically uses ATP as the energy source, and plays a key role during exercise such as in muscle contractions, control of blood flow, and oxygen delivery to skeletal muscles^31,32^. Collectively, these data suggest that the GMPS gene involves in the activity of the glutamine hydrolysis function by catalyzing XMP amination to produce GMP. Indeed, in the present study, we found that the young female subjects with the GMPS rs819865 G/A allele showed significantly higher association with cardiorespiratory endurance. Taken together, these data suggest that the GMPS-associated and ATP-involved amination reaction play a critical role for skeletal muscle function during exercise in humans and is a possible explanation for the relation between the GMPS rs819865 polymorphism and CRF and endurance performance.

The gene encoding human collagen XVIII (COL18A1), which included 43 exons and two promoters, has been mapped in the region 21q22.3^33^. This protein is the precursor of endostatin, a 20-kDa protein derived from the carboxy-terminal proteolytic fragment of collagen XVIII. Endostatin is a broad-spectrum angiogenesis inhibitor and interferes with growth factors such as VEGF. The VEGF gene is essential for promoting angiogenesis^34^. Breen et al. reported that the VEGF maintained capillary supply and apoptosis in normal skeletal muscles^35^. Amaral et al. suggested that VEGF was involved in angiogenesis induced by short-term exercise training, resulting in increased vascular density in skeletal muscle after exercise^36^. Exercise-induced angiogenesis increases the capillary area of O^2^ diffusion and shorten the diffusional distance of O^2^ from the capillary to the mitochondria^37^. In our results, the A/C allele of the COL18A1 gene was significantly associated with the 800-meter running performance in young female participants. Accordingly, these findings suggest that the COL18A1 rs117828698 polymorphism possibly results in increased biological activity of capillary number and apoptosis, vascular density, capillary area and distance of O^2^ diffusion in skeletal muscle, which is helpful to improve aerobic capacity and endurance performance.

The SNP rs79806428 is located in the PRKCA gene. Variants of the PRKCA have been found to be closely related to cardiac contractility and associated with cardiovascular disease^38,39^. Results from rodent and human studies indicated that PRKCA levels were inversely related to cardiac performance. Ventricular performance of mice lacking the PRKCA is enhanced, but cardiac performance of mice overexpressing the PRKCA by transgenic technology is impaired^40^. Overexpression of the PRKCA is also found in the patients with dilated cardiomyopathy^41^. At present, there is no research on the relationship between exercise and the PRKCA gene. Importantly, in this study we identified the C/T allele of the significant SNP, the PRKCA gene rs79806428, to be associated with cardiorespiratory fitness and endurance performance in young female non-athletes. Thus, in view of the positive effect of exercise on cardiac function, the PRKCA can possibly be used as a biomarker to study the relationship between exercise and cardiac function and rehabilitation in the future.

In this study, functional analyses were conducted in combination with the genome-wide association threshold to provide potential insights of mechanism for the identified associations. The eight SNPs, with four significant GWAS loci (GCOM1 rs17240160, GMPS rs819865, COL18A1 rs117828698, and PRKCA rs1951850) and four suggestive loci (AACSP1 rs12518860, CNTN5 rs1384206, RARG rs941138, PTPRD rs1951850) were identified to be associated with cardiorespiratory endurance. They are most likely to have functions associated with heart, lung, brain, and muscle tissues. Among the identified loci, the GMPS and COL18A1 genes are closely related to skeletal muscle, which is negatively correlated with CRF throughout life^42,43^. Several clinical studies have reported that patients with cardiovascular diseases experience a marked loss of skeletal muscle mass, may have reduced CRF, and consequently are exposed to higher risk of early mortality^44,45^. Thus, improving both CRF and muscle strength, but not either of the two, is the most effective behavioral strategy to reduce the risk of cardiovascular and all-cause mortality. Therefore, four identified significant SNPs (rs17240160, rs819865, rs117828698, and rs1951850) in the present study may provide new evidence and perspectives for future research on CRF and endurance performance. In addition, four suggestive SNPs (rs12518860, rs1384206, rs941138, and rs1951850) were also identified to be associated with CRF, though their P value did not reach the statistical threshold of genome-wide significance. Nonetheless, these data are very interesting, and it suggests that it could generate useful information if research could be expanded to multi-center and perhaps international efforts.

The present study has several limitations. Our statistical power is limited by the relatively small sample size (n = 762). However, our study subjects were restricted to female young individuals of Chinese Han ethnicity. It would avoid the interference of gender, age, and race in the results and allow a better depiction of the association of CRF and the identified loci. Thus, further replication and functional studies are necessary to confirm the present findings, especially in increasing sample size, adding males and exploring different age. In addition, the 800-meter running test was conducted only once. Indeed, we strictly followed the National Student Physical Fitness Standard to conduct the test and every participant was encouraged ensuring that the test results were their best performance. However, cautions should be taken because the nature of one-time observational study would affect its generalization of the results. Again, using an 800-meter running field test but not a direct measure of VO_2max_ would also affect validity of the results. Nevertheless, further innovative approaches and more powerful and comprehensive investigations are warranted to determine their worthiness and advance in the field of the exercise genomics of cardiorespiratory endurance phenotypes.

In summary, this study investigated the association between multiple single nucleotide polymorphisms and CRF and endurance performance in Chinese young female non-athletes. Our study is very likely the first GWAS on the association for this population. We found four significant loci (GCOM1 rs17240160, GMPS rs819865, COL18A1 rs117828698, and PRKCA rs1951850) were associated with CRF and endurance performance. The A/T allele of the GCOM1 rs17240160 polymorphism has more of a protective effect in increased activities of cardiovascular and neural protections. The GMPS rs819865 G/A allele and the COL18A1 rs117828698 A/C genotype play a critical role for skeletal muscle activity and function, which is relevant to CRF as indicated in previous studies. The PRKCA rs79806428 C/T allele has a significant association with cardiorespiratory endurance. Our results provide further genetic evidence for the polygenetic nature of cardiorespiratory endurance, which are also known to function in the heart, lung, brain, and muscle tissues. The identified four loci may be used as genomic biomarkers involved in cardiorespiratory endurance and intermediary traits of fitness and its trainability.

## Materials and Methods

### Participants

To avoid the influence of gender and ethnicity on the results, this study only recruited female college students (18-20 years, n = 800) who were Chinese Han ethnicity from Shanghai Jiao Tong University. Of those recruited 800 volunteers, 38 were finally excluded because of being unable to complete the test or meet the test requirement, such as performing the test without all-out-effort by walking and slow jogging. None of them were athletes. All participants were healthy and there were no apparent physical problems or pre-existing conditions. Written informed consent was obtained from each subject and all completed a health survey before the test. This study was approved by the Ethics Committee of BIO-X Institutes of Shanghai Jiao Tong University. All methods were performed in accordance with the relevant guidelines and regulations.

### 800-metre running test

Cardiorespiratory endurance is one important component of fitness assessment for college students implemented in China. The test involved running 1000 m for boys and 800 m for girls in accordance with fitness assessment of the Chinese National Student Physical Fitness Standard (CNSPFS) battery^18^. The subjects were requested to perform an 800-meter running test for CRF assessment. The 800-meter run is an extremely demanding event that requires substantial contributions from both the aerobic and anaerobic system, due to the high relative values of VO_2_ and high blood lactate concentrations attained after the run^19,20^. The validity of the test for Chinese female college students has been assessed previously^21^. All subjects performed the test on the each weekend in April, 2018 (temperature of 17.4 ± 6.8 °C) on the same 400-meter outdoor track and field. Prior to the test, participants jogged for 5 min as warm-up. Each participant was asked to complete the running test as fast as possible with all-out effort. The performance time was recorded in seconds as soon as completing the 800-meter run. The test procedures and requirements followed the CNSPFS Guidelines^18^. All tests were conducted by the qualified exercise professionals.

### DNA extraction

Five milliliters of venous blood were collected in tubes containing ethylenediaminetetraacetic acid (EDTA). Genomic DNA was extracted using a Quick Gene Model 610 L automated nucleic acid (DNA/RNA) extraction system (Fujifilm, Tokyo, Japan). It was diluted to a concentration of 50 ng/ml for SNP chip genotyping.

### Genotyping and quality control

Genome-wide genotyping was performed using the Illumina 1 M Array (Illumina Inc., San Diego, CA, USA) (data set 1) and Affymetrix Axiom CHB Array (Affymetric, Inc., Santa Clara, CA, USA) (data set 2). Samples were excluded if (1) the genotyping call rate was <95%, (2) heterozygosity was excessively low or high, (3) if the participants were not female or had ambiguous sex, and (4) were duplicates or relatives. Duplicates and close relatives were identified using PLINK’s identity-by-descent analysis. When a pair of samples had PI_HAT > 0.2, the member of the pair with the lower call rate was excluded from the analysis.

SNPs were excluded if MAF < 3% and call rate < 95% or in case of deviations from the Hardy-Weinberg equilibrium (P < 10^-5^). After quality control, 662,858 SNPs of 320 individuals (data set 1) and 1,057,935 SNPs of 442 individuals (data set 2) were retained for genotype imputation.

### Genotype imputation

Imputation was performed separately for each genotyping project data set. Genotypes were imputed into the reference panel from the 1000 Genomes Project^22^. A two-step procedure, involving pre-phasing in the first step and imputation of the phased data in the second step, was initially used to improve the computation efficiency. Pre-phasing was conducted using SHAPEIT^23^ and the subsequent imputation was performed using IMPUTE2^24^. Imputation was performed for each 5-Mb chromosome interval. Variants with INFO > 0.8, MAF > 0.03, a call rate ≥ 95%, and HWE P ≥ 1 × 10^−5^ were retained for further analysis. Variants presented in both data sets were saved for analyzing the association and meta-analysis. A set of 5,149,327 genetic variants for 762 individuals was retained for the final analysis.

### Statistical analysis

Baseline characteristics of measurements were expressed in terms of mean ± standard deviation (SD). Linear regression analysis was applied to investigate the associations between genetic variants and CRF in terms of 800-meter running time, which were adjusted by BMI^25,26^. The association and fixed-effects inverse variance-weighted meta-analysis were performed using PLINK^27^. A P value <5 × 10^-8^ was considered to have statistically genome-wide significance. A quantile-quantile (Q-Q) plot, generated using WGAViewer, was used to evaluate the overall significance of the GWAS results and the potential impact of population stratification.

## Supplementary information

Supplementary Information.
