# Supplementary material for: Four Loci Are Associated with Cardiorespiratory Fitness and Endurance Performance in Young Chinese Females
Source: Sci Rep. 2020 Jun 22;10:10117. doi: 10.1038/s41598-020-67045-y (PMC7723046; doi:10.1038/s41598-020-67045-y)
Supplement: Supplementary file 1 — Supplementary Information. [file 41598_2020_67045_MOESM1_ESM.docx]

Four Loci Are Associated with Cardiorespiratory Fitness and Endurance Performance in Young Chinese Females

Ying Zhao^1*^, Guoyuan Huang^2*^, Zuosong Chen^1^, Xiang Fan^1^, Tao Huang^1^, Jinsheng Liu^3^, Qing Zhang^4^, Jingyi Shen^4^, Zhiqiang Li^4,5,6#^ and Yongyong Shi^4,6,7,8#^

^1^ Physical Education Department, Shanghai Jiao Tong University, Shanghai 200240, China

^2^  Pott College of Science, Engineering and Education, University of Southern Indiana, Indiana 47712, USA

^3^ School Infirmary, Shanghai Jiao Tong University, Shanghai 200240, China

^4^ Bio-X Institutes, Key Laboratory for the Genetics of Developmental and Neuropsychiatric Disorders (Ministry of Education), Collaborative Innovation Center for Brain Science, Shanghai Jiao Tong University, Shanghai 200240, China

^5^ Affiliated Hospital of Qingdao University, Qingdao 266003, China

^6^ Biomedical Sciences Institute of Qingdao University (Qingdao Branch of SJTU Bio-X Institutes), Physical Education Department Qingdao University, Qingdao 266003, China

^7^ Shanghai Key Laboratory of Psychotic Disorders, Shanghai Mental Health Center, Shanghai Jiao Tong University School of Medicine, Shanghai 200030, China

^8^ Department of Psychiatry, First Teaching Hospital of Xinjiang Medical University, Urumqi 830046, China

***** Co-first-authors contributed equally to this work.

^#^ Correspondence: [lizqsjtu@163.com](mailto:lizqsjtu@163.com); [shiyongyong@gmail.com](mailto:shiyongyong@gmail.com)


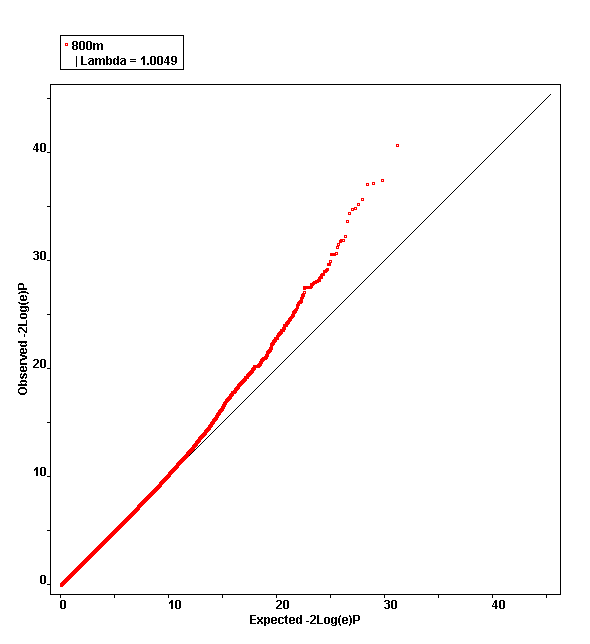


**Figure S1.** The QQ plot for the distribution of P values. Meta-analysis revealed four loci that reached the genome-wide significance level of *P* <5ⅹ10^-8^. These included four loci at *GCOM1* (rs17240160, *P*=1.73×10^-9^), *GMPS* (rs819865, P=8.56×10^-9^), *COL18A1* (rs117828698, *P*=9.59×10^-9^), and *PRKCA* (rs79806428, *P*=3.85×10^-8^) (Table S1). Among these SNPs, the top one was rs17240160 located near GCOM1 with a *P* value of 1.73×10^-9^ (Fig. S1). In addition, we found four suggestive SNPs that showed P<1×10^-7^, but did not achieve the genome-wide significance threshold.

Table S1. Results for heterogeneity analysis

| CHR | BP | SNP | Q | I |
| --- | --- | --- | --- | --- |
| 3 | 155716421 | rs819865 | 0.422 | 0 |
| 5 | 178208844 | rs12518860 | 0.329 | 0 |
| 9 | 9858194 | rs1951850 | 0.646 | 0 |
| 11 | 98243231 | rs1384206 | 0.375 | 0 |
| 12 | 53614349 | rs941138 | 0.125 | 57.46 |
| 15 | 58078261 | rs17240160 | 0.860 | 0 |
| 17 | 64278885 | rs79806428 | 0.053 | 73.21 |
| 21 | 46849501 | rs117828698 | 0.197 | 39.97 |

Note: Q = p-value for Cochrane's Q statistic; I = I^2 heterogeneity index (0-100).

**Table S2**. LD for the genome-wide significant variants with their nearby variants (distance<500kb) with P<1e-4.

| CHR_A | BP_A | SNP_A | CHR_B | BP_B | SNP_B | r^2^ |
| --- | --- | --- | --- | --- | --- | --- |
| 3 | 155716421 | rs819865 | 3 | 155632019 | rs2320318 | 0.849 |
| 3 | 155716421 | rs819865 | 3 | 155634623 | rs76182393 | 0.849 |
| 3 | 155716421 | rs819865 | 3 | 155706888 | rs819851 | 0.914 |
| 3 | 155716421 | rs819865 | 3 | 155707288 | rs118026643 | 0.811 |
| 3 | 155716421 | rs819865 | 3 | 155716421 | rs819865 | 1.000 |
| 15 | 58078261 | rs17240160 | 15 | 58070608 | rs12914158 | 0.266 |
| 15 | 58078261 | rs17240160 | 15 | 58071175 | rs35646184 | 0.283 |
| 15 | 58078261 | rs17240160 | 15 | 58077035 | rs2414520 | 0.380 |
| 15 | 58078261 | rs17240160 | 15 | 58078261 | rs17240160 | 1.000 |
| 17 | 64278885 | rs79806428 | 17 | 64233229 | rs16958984 | 0.355 |
| 17 | 64278885 | rs79806428 | 17 | 64236042 | rs10048208 | 0.355 |
| 17 | 64278885 | rs79806428 | 17 | 64238830 | rs8072732 | 0.355 |
| 17 | 64278885 | rs79806428 | 17 | 64239125 | rs112412429 | 0.355 |
| 17 | 64278885 | rs79806428 | 17 | 64239709 | rs7221173 | 0.355 |
| 17 | 64278885 | rs79806428 | 17 | 64240013 | rs16959000 | 0.355 |
| 17 | 64278885 | rs79806428 | 17 | 64242068 | rs62073345 | 0.359 |
| 17 | 64278885 | rs79806428 | 17 | 64242351 | rs112502566 | 0.359 |
| 17 | 64278885 | rs79806428 | 17 | 64244070 | rs8071678 | 0.359 |
| 17 | 64278885 | rs79806428 | 17 | 64278885 | rs79806428 | 1.000 |
| 21 | 46849501 | rs117828698 | 21 | 46846842 | rs117873808 | 0.842 |
| 21 | 46849501 | rs117828698 | 21 | 46848320 | rs13046232 | 0.809 |
| 21 | 46849501 | rs117828698 | 21 | 46849501 | rs117828698 | 1.000 |
| 21 | 46849501 | rs117828698 | 21 | 46849651 | rs2838930 | 0.984 |
| 21 | 46849501 | rs117828698 | 21 | 46849749 | rs7275991 | 0.967 |
| 21 | 46849501 | rs117828698 | 21 | 46849777 | rs17004772 | 0.984 |
| 21 | 46849501 | rs117828698 | 21 | 46850925 | rs115491384 | 0.919 |
| 21 | 46849501 | rs117828698 | 21 | 46852247 | rs139136602 | 0.872 |
| 21 | 46849501 | rs117828698 | 21 | 46852758 | rs114065856 | 0.872 |
| 21 | 46849501 | rs117828698 | 21 | 46871472 | rs2838932 | 0.246 |

**Table S3.** Functional implications of the identified loci.

| Study ID | Paper Title | PMID | Tissue | Correlated Gene | p-value |
| --- | --- | --- | --- | --- | --- |
| GTEx2015_v6 | The Genotype-Tissue Expression (GTEx) pilot analysis: Multitissue gene regulation in humans | [25954001](http://pubmed.gov/25954001) | Cells_Transformed_fibroblasts | C3orf33 | 6.299E-07 |
| GTEx2015_v6 | The Genotype-Tissue Expression (GTEx) pilot analysis: Multitissue gene regulation in humans | [25954001](http://pubmed.gov/25954001) | Whole_Blood | COL18A1 | 7.845E-07 |
| GTEx2015_v6 | The Genotype-Tissue Expression (GTEx) pilot analysis: Multitissue gene regulation in humans | [25954001](http://pubmed.gov/25954001) | Muscle_Skeletal | CEP112 | 2.380E-08 |
